# Efficacy of the Small Step Program in a Randomized Controlled Trial for Infants under 12 Months Old at Risk of Cerebral Palsy (CP) and Other Neurological Disorders

**DOI:** 10.3390/jcm8071016

**Published:** 2019-07-11

**Authors:** Linda Holmström, Ann-Christin Eliasson, Rita Almeida, Catarina Furmark, Ann-Louise Weiland, Kristina Tedroff, Kristina Löwing

**Affiliations:** 1 Paediatric Neurology, Department of Women’s and Children’s Health, Karolinska Institutet, SE 171 77 Stockholm, Sweden; 2 Stockholm University Brain Imaging Centre (SUBIC), Stockholm University, SE 106 91 Stockholm, Sweden; 3 Department of Neuroscience, Karolinska Institutet, SE 171 77 Stockholm, Sweden; 4 Neuropediatric Department, Astrid Lindgren Children’s Hospital, Karolinska University Hospital, SE 171 76 Stockholm, Sweden

**Keywords:** early intervention, cerebral palsy, development, other neurological disorder, gross motor function, upper limb function, communication

## Abstract

The objective was to evaluate the effects of the Small Step Program on general development in children at risk of cerebral palsy (CP) or other neurodevelopmental disorders. A randomized controlled trial compared Small Step with Standard Care in infants recruited at 4–9 months of corrected age (CA). The 35-week intervention targeted mobility, hand use, and communication during distinct periods. The Peabody Developmental Motor Scales^2ed^ (PDMS-2) was the primary outcome measure. For statistical analysis, a general linear model used PDMS-2 as the main outcome variable, together with a set of independent variables. Thirty-nine infants were randomized to Small Step (*n* = 19, age 6.3 months CA (1.62 SD)) or Standard Care (*n* = 20, age 6.7 months CA (1.96 SD)). Administering PDMS-2 at end of treatment identified no group effect, but an interaction between group and PDMS-2 at baseline was found (*p* < 0.02). Development was associated with baseline assessments in the Standard Care group, while infants in the Small Step group developed independent of the baseline level, implying that Small Step helped the most affected children to catch up by the end of treatment. This result was sustained at 2 years of age for PDMS-2 and the PEDI mobility scale.

## 1. Introduction

The effects of various early intervention programs for children at risk of cerebral palsy (CP) or other neurodevelopmental disorders have been recognized as an important emerging research field in recent years. Early intervention programs allow utilization of the developmental “window of opportunity” presented by the activity-dependent plasticity and rapid development of the central nervous system during the first years of life. To exploit this potential, there is a need to identify children at risk of CP and other neurological disorders at a very early age [1,2]. Although neurological signs can be present during the first months of life, it has traditionally been recommended that a diagnosis such as CP should not be made until later [3,4]. Neurodevelopmental disorders such as autism spectrum disorder are typically diagnosed even later, and rarely before three years of age [5]. A group of children that could also benefit from early intervention programs, and are more easily identified, are those born extremely prematurely. Most such children do not develop CP but may still need interventions due to deficits in motor and other skills following preterm birth [6,7]. If an early intervention program applies a functional skill learning perspective but without focus on diagnosis or specific conditions, we think the children at high risk, displaying neurological signs early in life, can likely be included. 

Recent systematic reviews suggest that a combination of interventional elements, based on newly developed theoretical frameworks, should be included in future early intervention programs. Evidence is still very limited for the effects of intervention on motor development in infants at high risk of CP [8,9], but tentative positive results following early intervention have been found for communication and cognitive development as well as hand motor function in children with CP [10,11,12,13]. For infants born prematurely, early interventions seem to have more positive effects on cognition than on motor development when followed up until school age [14]. In addition, reduced parental stress and facilitated child–parent bonding have been reported as a result of early intervention [15,16]. Given these positive findings in different developmental areas, it is reasonable to assume that infants with a history of neonatal events and at risk of future diagnoses such as CP, neurodevelopmental disorder, and delayed development could benefit from intervention. 

This study evaluates the effects of the recently developed Small Step Program for infants at risk of developing CP and other neurodevelopmental disorders. Small Step was designed to include a combination of intervention elements to provide individualized, goal-directed, and intensive intervention addressing three distinct foci (i.e., hand use, mobility, and communication) provided in the child’s own home environment and conducted by their parents, who were trained and coached by therapists, and to be used from 4 months of age. The theoretical background, rationale, contents, and structure of the Small Step Program have previously been extensively described in a methods paper [17] and the protocol is registered at ClinicalTrials.gov. 

We hypothesize that a treatment period with the newly developed Small Step Program, for 35 weeks in the first year of life, will have positive effects on various aspects of child development, effects that exceed those of Swedish Standard Care in children at high risk of CP or other neurodevelopmental disorders. The primary objective was therefore, first, to evaluate the effects of Small Step on motor development using the Peabody Developmental Motor Scale (PDMS-2) as the primary outcome measure and, second, to investigate how various child and family characteristics might influence this outcome.

## 2. Methods and Design

**Figure 1:**
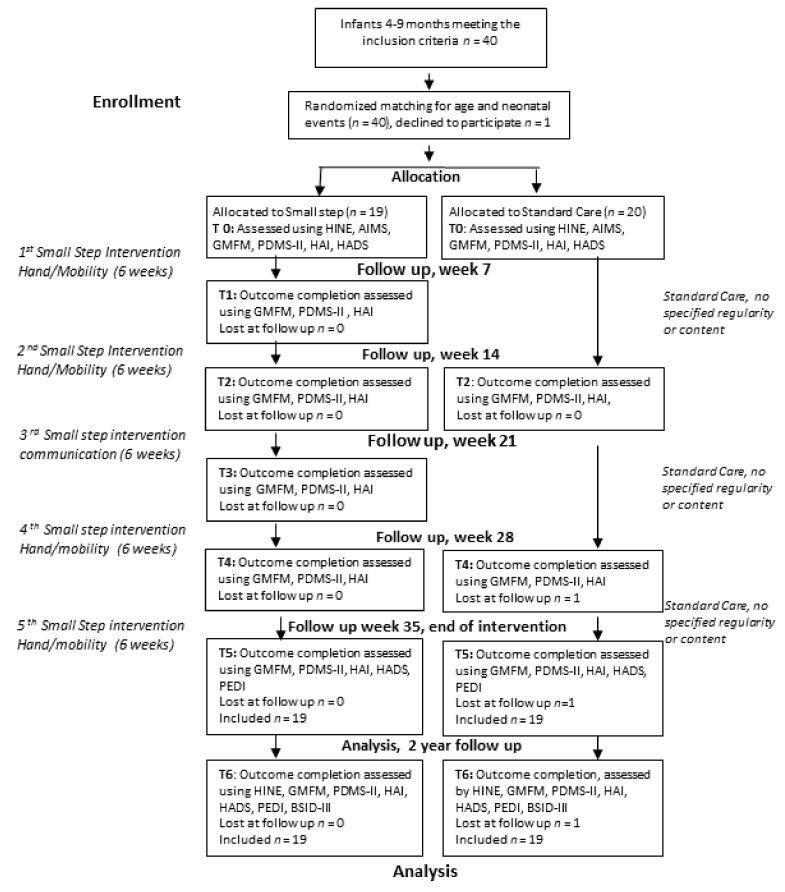
Flow diagram of data collection procedure.

The design was a prospective, randomized, controlled trial (RCT). The trial had two arms, i.e., the Small Step Program and Standard Care, and was conducted from 2014 to 2018 at the Astrid Lindgren Children’s Hospital, a tertiary hospital in Stockholm, Sweden. Participation in the study did not affect the general care of the infants, and all other medical interventions continued as planned for both groups. All parents received oral and written information after baseline assessment but before randomization, allowing them to consider their decision before giving oral and written informed consent. The study followed the Consolidated Standards of Reporting Trials (CONSORT) and was approved by the Stockholm Regional Ethical Review Board (no. 2013/2044-31/1). The ClinicalTrials.gov registration number is NC2166801, registered 12 June 2014, and further details are presented in the study protocol [17]. The study period was 35 weeks, with repeated assessments conducted during this period and at the follow-up when the children were 2 years of age (Figure 1).

### 2.1. Participants

Infants at risk of CP or other neurodevelopmental disorders were recruited from the regular neonatology standardized clinical follow-up program at about 3 months of corrected age (CA, i.e., corrected for prematurity) or at the pediatric neurology clinic. The infants had typically been exposed to perinatal risk factors such as preterm birth, hypoxia, infections, heart insufficiency, small for gestational age/growth restrictions, hypoxic ischemic encephalopathies (HIEs), or presented with morphological brain abnormalities. Inclusion criteria, based on the information available in the hospital, were 4–9 months CA and neurological signs based on a combination of assessments, such as the Hammersmith Infant Neurological Examination (HINE) [18] and the Alberta Infant Motor Scale (AIMS, using 2 SD as the cut-off) [19,20], together with the findings of other clinical neurological examinations. Magnetic resonance imaging (MRI) findings were used as a basis for inclusion when available. Exclusion criteria were unstable health, uncontrolled epilepsy, a progressive condition, or neither parent being fluent in Swedish.

### 2.2. Randomization, Blinding, and Sample Size

The baseline assessments of the children were conducted before randomization. Randomization was based on a prepared random assignment number list. The children were allocated to groups by block randomization, with each block including four slots. Stratification was used to control for gestational age (preterm <37 weeks and term >37 weeks), because gestational age is known to affect development. Children assigned to the Small Step Program were further randomized to start with either the mobility or the hand use intervention. An infant was assigned to the next free slot on the list by the principal investigator (PI). The list was kept in a locked space only available to the PI, who was not involved in recruitment. Neither the families nor the therapists responsible for data collection were blinded to group allocation. The therapists responsible for data collection at each assessment point were not involved in the intervention, were unbiased as to group allocation, and had no access to previous assessments. The study sample size was estimated from the data on effects presented in the pilot project of Morgan et al. [9]. A power calculation was performed using the motor composite scores of the Peabody Developmental Motor Scale (PDMS-2), with an alpha value of 5%, power of 80%, a minimal clinically important difference of 10%, and a 20% drop-out rate, resulting in a total of 30 participants (15 per group) [21]. 

### 2.3. Intervention

The Small Step Program had three intervention foci: mobility, hand use, and communication. Altogether, the program had five steps, the hand use and mobility foci being performed twice (starting in a randomized order, see Figure 1) and the communication focus once. Each step lasted six weeks and included six home visits by therapists for hand use and mobility and four home visits for communication. The parents were expected to be the training providers on a daily basis, supported by coaching and supervision from the therapists responsible for the specific focus area. Before and after each step, the families were asked to come to the hospital for assessments.

The rationale for dividing the intervention program into three foci was to help the families learn more about different areas of development and to optimize the training within each of these areas. The general principles of all foci were: to assume that children have an inner drive to explore their environment, to assume that it is important to identify the child’s strengths, and to use short-term goals for practice (i.e., what to focus on in the upcoming training week). In all foci, the goals were set through collaboration between the parents and the responsible therapist. The parents were helped to determine what their infant was likely to learn in the next step, in light of the child’s present functional and cognitive level. The defined goals were written in the program diary kept by the parents. The goals were formulated as activities meaningful in the everyday life of the child and family. Great emphasis was put on the children’s self-initiated actions, which were stimulated by meaningful, motivating, challenging, and playful activities and toys. 

For the hand use focus, the assumption was that grasping abilities help infants explore their environment. Interesting toys chosen to suit the child’s developmental level and level of hand function stimulated the child to perform self-initiated actions. Object exploration triggered different hand actions and stimulated cognitive development. In the mobility focus, the aim was to help the child learn new gross motor activities that would allow them to explore their environment. The training was tailored to the child’s prerequisites and motivation, and included activities such as: maintaining and changing the body position, sitting, standing, crawling, and moving. The communication focus was based on the assumption that children have an inner drive to communicate. The intervention was directed towards encouraging families to be aware of the child’s subtle signs of intention to communicate. A child-centered approach was applied by actively observing the moment-by-moment focus of the child, to promote interactions by following the child’s lead by imitating, interpreting, and expanding the child’s vocalizations and non-verbal signals (i.e., gazes, gestures, and bodily movements). Further details can be found in the study protocol [17]. 

Standard Care was provided for children in the control group. They received intervention from physiotherapists at the hospital and parents were given advice on home training. The families typically visited the physiotherapist once a month until the children were referred to the rehabilitation services at some point during their first year of life. The children continued to receive interventions from physiotherapists and other team members when needed. The frequency of Standard Care interventions was not standardized but was set to meet the child’s individual needs within a large range of variation. The interventions occurred at the rehabilitation center or the home. The treatment was based on family-centered intervention and functional training. In this study, the dosage of standard care was calculated based on the number of appointments noted in the medical records, but did not include the additional appointments at the hospitals that were part of the study. 

### 2.4. Data Collection Procedure

Children and parents in both groups were assessed at multiple follow-ups at the hospital (Figure 1). Only data from baseline (T0), end of intervention (T5), and when the child was 2 years old (T6) are presented here.

### 2.5. Primary Outcome Measure

PDMS-2 is a standardized measure assessing gross and fine motor skills in young children from birth through 5 years of age [22]. Of the six subtests, the stationary, locomotion, grasping, and visual motor integration subtests were used, and the raw scores were analyzed (we did not find the reflex subtest to be relevant and the object manipulation subtest has no item for children below 12 months of age); for further details, see the section “Statistics”.

### 2.6. Secondary Outcome Measure

For children, we used Gross Motor Function Measure-66 (GMFM-66), an observational, standardized, and criteria-referenced measure evaluating changes in gross motor function in children with CP, from 5 months of age. The items are organized in order of increasing difficulty, ranging from 0 (low capacity) to 100 (high capacity) [23,24]. Hand Assessment for Infants (HAI) is an observational, criteria-referenced measure of upper limb and hand use from 3 months of age in infants at risk of developing CP; it ranges from 0 (low capacity) to 100 (high capacity) [25]. 

For parents, we used the Hospital Anxiety and Depression Scale (HADS), a self-report scale developed to detect states of depression and anxiety [26]. The scale is divided into two subscales, depression and anxiety, comprising seven items each, with ratings ranging from 0 to 3. Each subscale is summarized. On an individual level, a score above 10 on either scale is considered indicative of a clinical condition, while scores of 7–10 are considered mild to moderate. In this study, we used a cut-off of 8 when reporting individual results. The families were expected to complete the questionnaire at home and return it in a prepaid envelope.

### 2.7. Follow-up Outcomes at Two Years of Age 

In addition to previously used assessments, the following assessments were added at 2 years of age. Bayley Scales of Infant Development (BSID-III) is a standardized measure assessing receptive and expressive language development, cognitive development, and fine and gross motor development in infants and toddlers aged 0–3 years [27]; raw scores were analyzed. The Pediatric Evaluation of the Disability Inventory (PEDI) is a standardized interview evaluating functional skills in the domains of self-care, mobility, and social function in children aged 6 months to 7.5 years [28]. A Swedish version was used [29]. Scaled scores, ranging from 0 to 100, were used for analyses. A questionnaire assessing feasibility and acceptability was sent out after the last appointment; all parents were asked to answer six questions regarding study participation, and parents in the Small Step Program were asked to answer an additional four questions specific to participation in the intervention group.

### 2.8. Diagnosis and Brain Pathology

Clinical MRI was used to investigate the general type of brain lesion characteristics [4]. If clinically justified, MRI was performed during the children’s first year as part of their routine care. The MRI results were interpreted by a neuroradiologist unaware of the infants’ clinical diagnosis, other outcomes, and group allocation in the study. To estimate the neurological status of the infants, HINE was used before inclusion as well as at later time points to check for the presence of or change in neurological signs [18]. In HINE, the overall score ranges from 0 to 78. Typically developing infants have three- and six-month median scores equal to or greater than 67 and 70, respectively.

Diagnosis was determined at 2 years of age (CA) by a child neurologist (KT) during an extended visit. Diagnosis was based on the medical history provided by parents, clinical findings, data from medical records, and MRI results when available. When appropriate, one or several diagnoses were identified. For the diagnosis and subtype of CP, the Surveillance for Cerebral Palsy in Europe criteria [4] were applied. All children were further classified according to two five-level ordinal classifications for children with CP: the Gross Motor Functional Classification System, Expanded & Revised (GMFCS-E&R), emphasizing performance in sitting, walking, and wheeled mobility [30], and the Mini Manual Ability Classification System (Mini-MACS), classifying how children below 4 years of age handle objects in daily life [31]. All children, irrespective of diagnosis, were classified according to these systems.

### 2.9. Statistics

Analysis was conducted using R, version 3.2.3. Descriptive statistics, frequencies, means, and SD or confidence intervals were used to describe the data. Data at time point (T) 0 were checked for group differences. Continuous variables were controlled for normality using the Shapiro–Wilk test. Variables that were not normally distributed were compared across groups using the Wilcoxon rank sum test. Variables that did not significantly differ from normality were compared using a two-sample *t*-test. Categorical variables were compared using Pearson’s chi-square test with a simulated *p*-value based on 10,000 simulations. 

The primary outcome measure comprised scores on four subscales of PDMS-2. Pearson correlation between the subscales across participants was calculated at both T0 and T5. Given that the subscales were highly correlated (see “Results”), and for simplicity, a single PDMS-2 raw score was created for each subject by averaging the subscale scores.

A general linear model was used with mean (m) PDMS-2 at T5 as the outcome (dependent) variable. The following measures were used as possible explanatory (independent) variables: mPDMS-2 at T0, group (Small Step or Standard Care), interaction between mPDMS-2 and group, gestational age, age in months at inclusion, hours of therapist-led treatment, time between T0 and T5, basic pattern of brain injury (MRI), HINE, and HADS (total mean). MRI results were described using a categorical variable with the following classes: no visually detected problems, maldevelopment, white matter deficits, grey matter deficits, and other problems. Other included (independent) variables were GMFCS-E&R, interaction between GMFCS-E&R and group, and diagnoses. GMFCS-E&R was considered a binary variable with one level corresponding to level I or II, and the other level corresponding to levels III–V (GMFCS-E&R/2). Diagnosis was also considered a binary variable with the two levels corresponding to the presence or absence of a CP diagnosis.

Additionally, several other variables were used as the outcome variables of a similar general linear model, using the same explanatory variables. The other variables used as outcomes were: the secondary outcomes GMFM-66 and HAI at T5; and mPDMS-2, BSDI-III, and PEDI at T6. The significance level was set at *p* < 0.05.

## 3. Results

### 3.1. Participants

Forty infants from 38 families were recruited. One family was lost after randomization due to dissatisfaction with allocation. Nineteen infants were randomized to the Small Step Program and 20 to Standard Care. One set of twins was included in each group. One infant dropped out of Standard Care at T2 because the family chose to discontinue participating in the study. All analyses using data for infants past T0 therefore include 19 infants in each group (Figure 1). For parental outcomes, we lack HADS data from five mothers at T0, nine at T5, and 15 at T6 because the families did not return the questionnaires, despite being reminded three times. The infants’ mean age at inclusion was 6.3 months (SD 1.62) and 6.7 months (SD 1.96) for the Small Step and Standard Care groups, respectively. The length of inclusion in the study varied somewhat, due to sickness or everyday difficulties attending the appointments at the hospital for the interim assessments (T1–T4). For further demographic data, see Table 1. The data at baseline (T0) did not indicate any significant differences between the groups (*p* > 0.05) (Table 1). Descriptive results for the different outcome measures for the infants are presented in Table 2.

### 3.2. Primary Outcome Investigated after Intervention, T5

The scores of the four subscales of PDMS-2 were highly correlated across participants, at both T0 (pairwise correlations, 0.62–0.86) and T5 (pairwise correlations, 0.79–0.91). For simplicity, a single average score (mPDMS-2) was used for each time point (see “Methods and Design”).

A general linear model was used to explain the main outcome variable, i.e., mPDMS-2 after intervention (T5), based on the following variables: mPDMS-2 at T0, group, interaction between mPDMS-2 at T0 and group, age at inclusion, HINE at T0, time between T0 and T5, gestational age, hours of therapist-led treatment, GMFCS, and diagnosis. This model revealed a significant effect of the interaction between mPDMS-2 at T0 and group (*p* = 0.04). 

The variables gestational age and hours of therapist-led treatment were very far from significant. The variables diagnosis and GMFCS overlapped considerably. We therefore reduced the model by excluding the variables gestational age, hours of therapist-led treatment, and diagnosis. This final model (Table 3) was associated with a slightly better Akaike information criterion. The main conclusion remained qualitatively the same, namely, that there was a significant interaction between mPDMS-2 at T0 and group (*p* = 0.02).

**Figure 2:**
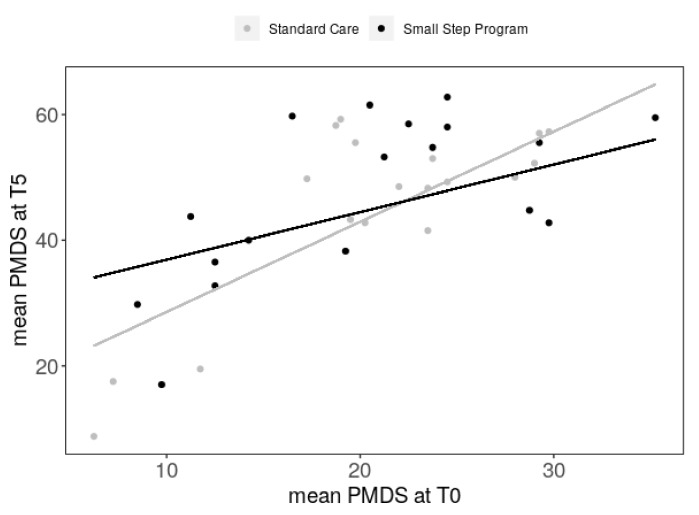
A general linear model showing that the group effect on mPDMS-2 after intervention (T5) depended on the baseline (T0) values (interaction, *p* = 0.02). Data are represented in black for Small Step and grey for Standard Care.

A significant interaction means that the effect of group depends on the mPDMS-2 level at T0. To interpret this result, the data were further analyzed separately for the Small Step and Standard Care groups, using the same explanatory variables. We found that mPDMS-2 at baseline (T0) was a significant predictor of the outcome after intervention (T5) in the Standard Care (*p* = 0.006) but not the Small Step group (*p* = 0.12, Figure 2). So, in the Small Step group, the outcome after intervention (T5) was not significantly explained by the level of performance at baseline (T0). These data suggest that children in the Small Step group, who started with poor performance at baseline (T0), caught up with children who started with better performance at baseline (Figure 2).

Additionally, we investigated whether the effect observed could instead be explained by an interaction between the children’s severity level and group. For this, we used a linear model incorporating all the previous variables as explanatory variables plus the interaction between group and GMFCS-E&R/2 level. This interaction was not significant (*p* = 0.25) and the main result remained qualitatively the same. Furthermore, we checked whether our main result remained the same in a model that instead incorporated GMFCS-E&R/2 and the children’s diagnosis (i.e., CP/no CP) defined at 2 years of age (T6), and the interaction between diagnosis and group. The interaction was not significant (*p* = 0.99) and the main result remained qualitatively the same.

Due to missing data for the variables neuroradiology (10 children) and HADS (9 mothers at T5), these were not included in the main analysis. However, we extended the main analysis to include these variables, one at a time, with no qualitative change.

### 3.3. Secondary Outcomes after Intervention, T5

A general linear model was also used to investigate secondary outcome measures. The model used was similar to the final model described above (Table 3), with GMFM-66 replacing PDMS-2 after intervention (T5) and at baseline (T0) and all other explanatory variables the same. There was a trend for the interaction between GMFM-66 at baseline and group to have an effect (*p* = 0.06). GMFM-66 at baseline (T0) was predictive of GMFM-66 after intervention (T5) in the Standard Care (*p* = 0.037) but not the Small Step group (*p* = 0.17). The severity level of GMFCS/2 was predictive of both Small Step (*p* = 0.004) and Standard Care results (*p* = 0.002). 

We used a similar model (Table 3) to study HAI after intervention (T5). PDMS-2 after intervention (T5) and PDMS-2 at baseline (T0) were replaced by HAI at T5 and at T0, respectively; all other explanatory variables in Table 3 were included. The interaction between HAI at baseline (T0) and group was not significant (*p* = 0.153). HAI at baseline (T0) was a predictor of HAI after intervention (T5, *p* = 0.0001). Severity according to GMFCS-E&R/2 was also a significant predictor of HAI after intervention (*p* = 0.048). 

### 3.4. Follow-up at Two Years of Age, T6

A similar analysis was conducted to model mPDMS-2 at the 2-year follow-up. All explanatory variables listed in Table 3 were used. The interaction between group and mPDMS-2 at baseline (T0) was still highly significant (*p* = 0.017). This means that the relationship between the baseline (T0) measure and the primary outcome PDMS-2 (T6) was still dependent on the group. 

We used models similar to those described above to investigate whether T0 scores affected other T6 outcomes in a differential way depending on the group. The following measurements were used as outcome variables in separate models: PEDI self-care, PEDI mobility, PEDI social function, BSDI-III motor, BSDI-III language, and BSDI-III cognitive index. The explanatory variables used were those in Table 3. For the PEDI mobility score, there was a significant interaction between group and mPDMS-2 at baseline (T0) (*p* = 0.023). Mean PDMS-2 at baseline (T0) still had an effect for Standard Care (*p* = 0.007), but not for Small Step (*p* = 0.10). The results were qualitatively the same when we used GMFM-66 instead of mPDMS-2 at baseline (T0). No significant effects were found for the other outcome measures considered.

### 3.5. Child Diagnostic Outcomes

At 2 years of age, 20 of 38 participants received a diagnosis of CP, *n* = 10 in the Small Step Program and *n* = 10 in Standard Care. One child in each group was diagnosed with autism spectrum disorder. Thirteen children were diagnosed with other neurodevelopmental disorders and two children in Small Step and one child in Standard Care displayed development within the typical or close to typical range and thus did not receive any diagnosis (Table 4 and Supplementary Tables S1 and S2). The proportions of epilepsy and high comorbidity were larger in the Small Step group (Table 4). The children with other neurodevelopmental disorders all scored below the age-normative score on mPDMS-2 (except for the grasping subscale) at 2 years of age (Supplementary Tables S1 and S2). More children were at level I of both GMFCS-E&R and Mini-MACS in the Standard Care group (Table 4). Almost all children with other neurodevelopmental disorders were classified at level I of GMFCS-E&R and Mini-MACS (Supplementary Tables S1 and S2).

### 3.6. Well-being of Mothers

The mother’s well-being was measured using HADS, which indicated that almost one-third of the 37 mothers scored above the suggested cut-off (i.e., 8) for clinical signs of anxiety (*n* = 11) and depression (*n* = 10) at baseline. Signs were much more frequent in the Small Step group (Table 1). The total mean HADS rose from 13.5 (SD 8.53) to 15.0 (SD 11.61, missing *n* = 3) in the Small Step group and from 10.35 (SD 7.0, missing *n* = 5) to 11.38 (SD 6.94, missing *n* = 6) in the Standard Care group from baseline (T0) over the course of the intervention (T5). The mean scores were somewhat higher in the Small Step group, but given the large number of missing data, this should be interpreted with caution.

### 3.7. Parental Experience of the Project

In general, the families were very satisfied to be included in the study, but parents in the Small Step group scored even higher (Table 5). The answers to specific questions about the characteristics of Small Step indicate that it was very well received. 

## 4. Discussion

There was no difference between the children who received the Small Step Program and those who received Standard Care in terms of their level of development at end of the intervention or at 2 years of age. However, the developmental level was influenced by the baseline status. In the Standard Care group, the infants who started with low ability improved less than did those who started somewhat higher, while infants in the Small Step group developed independent of their baseline level. This implies that Small Step helped the most affected children to catch up to the less affected children by the end of the treatment period, a result that was sustained at 2 years of age for PDMS-2 and the PEDI mobility scale. These results were robust when various models were tested to find the optimal statistical model. Diagnosis, CP or not, did not influence the results. The parents found the Small Step Program to be both feasible and acceptable. 

This interpretation, i.e., that the Small Step Program positively affected the lowest functioning infants, can be further understood from Table 4. The proportions of infants with high levels of comorbidity, epilepsy, and severe functional limitations (i.e., low GMFCS-E&R and Mini-MACS level) at 2 years of age were higher in the Small Step than Standard Care groups. All these factors are known to be limiting for child development [32]; however, despite these limitations, the infants caught up. This could be because the different aspects of Small Step helped parents to maximize their infants’ limited ability to self-initiate actions by training them in a variety of activities, ultimately helping the children realize more of their developmental potential. These results also support the importance of the coaching component for parents in the program. Improvements among more severely affected infants have not been found in previous early intervention programs, such as GAME and Baby CIMT, in which the less affected children benefited the most [11,13]. 

### 4.1. The Unique Concept and Feasibility of the Small Step Program

The Small Step Program was created based on recent knowledge of effective interventions for older children with CP, as described in the study protocol [17]. The uniqueness of Small Step as an early intervention program is its broad approach, addressing both hand skills and communication in addition to gross motor function. The program is also clear and specific within its different foci, with specific small achievable goals set in collaboration with parents, and strengthens the parental role by allowing parents to be in charge of the training situation. This broad, specific, and intensive approach has not been applied in the same way in any previously reported intervention program and was not used in the Standard Care group. In Swedish Standard Care, families typically meet physiotherapists on an ongoing but inconsistent basis depending on their needs and the available resources, but children in this age range are not given periods of intensive training. Families only occasionally meet other professionals in addition to the physiotherapist during the child’s first year of life. Although the Standard Care in Sweden is developing under the influence of new ideas, there was a pronounced difference between Small Step and Standard Care in how the different intervention components were emphasized, organized, and utilized.

The Small Step Program promotes development and general learning. It has no components specifically targeting CP, and we found the program beneficial for children with various neurodevelopmental disorders. Although unique, the Small Step concept has similarities to other recently developed early intervention programs, such as COPCA and GAME, which are all individualized and goal specific [13,33]. 

In the Small Step Program, with interventions organized in five steps and involving clinical experts from various professions, program acceptability was crucial. Acceptability was investigated by follow-up questions for parents asking about this periodicity and the exchanges with professionals. The parents were very satisfied with this arrangement, rating the program very high, i.e., above 8 on a 0–9 scale. This suggests that parents found that the different components, the structure in which only one component is trained at a time, and the exchanges with clinical experts concerning the focus areas facilitated beneficial parental learning about the child’s overall development. The feasibility of Small Steps was also shown by the absence of drop-outs from the program and by parents rating their motivation to engage in the program as very high (8.3 on a 0–9 scale).

### 4.2. Effect of Small Step in Relation to Previous Evidence

The knowledge gained from this study adds evidence regarding certain aspects of early intervention programs [8,9,14]. One can speculate as to why only limited results were achieved here: perhaps we were unable to create an effective treatment approach suitable for all included infants, or perhaps the early Standard Care provided for infants in Stockholm is good enough to promote development. Almost all included children were also referred to the rehabilitation service from an early age, during the period when they were part of the study, which is not typical. This might have influenced the results and confounded the effects of the Small Step Program. There was probably also some theoretical overlap between the Small Step Program and Standard Care. In Swedish rehabilitation services, Standard Care is based on family-centered intervention and functional training. However, intensive training applied and organized as in Small Step is not part of Swedish Standard Care. Swedish Care differs from what is commonly reported in other countries and cultures. For example, in the Netherlands, typical standard care is reportedly based on neurodevelopmental treatment (NDT) and in Australia an eclectic approach including both NDT and sensory integration is used [13,33]. Also, the frequency of Standard Care varies, and it seems to be more frequent in the Netherlands than Australia. In this study, we noted the total number of therapy-led interventions for the infants in Standard Care with the hospital/rehabilitation services. As expected, Standard Care involved fewer appointments than did Small Step, but the number of appointments was higher than is typical in Sweden, possibly because the Hawthorne effect influenced both the service providers and the families. The families included in Standard Care also had several data collection appointments (not included in Table 1) at the hospital as part of the study protocol; they very much appreciated these appointments and described them as meaningful in the follow-up questionnaire. In light of this and the non-significant difference in outcome between groups, the null hypothesis could not be rejected, i.e., we could not prove that Small Step is more effective than Standard Care.

### 4.5. Strengths and Limitations 

The results of early intervention programs will always be greatly influenced by the fact that it is difficult to accurately predict CP or other neurological disorders in infants and thereby select the “right” children for inclusion. We used a combination of assessments, all of which have limitations when used separately. For example, AIMS is not a diagnostic tool, but rather measures the motor development of infants at risk of motor delay, focusing on attaining motor milestones, and should be used with caution for preterm children who are known to be late developing but catch up at a later age. We lacked some data from MRI, which is not routinely used on all children at the clinic. For children at risk of CP, General Motor Assessment (GMA) is recommended at three months of age [2,34], but we could not use GMA, again because it is not part of the standardized follow-up program at hospitals in Sweden. Despite this, our inclusion criteria resulted in more than 50% children with CP, though this was less than was achieved in the GAME study [13]. Another limitation is of course the small sample size, which does not allow for further sub-analysis. The power analysis may not be precise, since we used different inclusion criteria and different intervention compared to the study we used for power calculation [21]. All included children benefited from the Small Step Program independent of diagnosis. Only three children seemed to manifest fairly typical development; the others, with various neurodevelopmental disorders, had still not reached the expected development in relation to age norms in PDMS-2 (see Supplementary Table S2). Yet, it is unknown whether children with different types of neurological impairments may respond differently to the treatment. The variety of diagnoses and the inclusion of three children without diagnoses might also have confounded the potential effect.

High practice intensity is part of the Small Step Program and has been proven to be an important element of effective training in older children [35,36]. We did not find it relevant to count the children’s training hours (the therapist-led intervention was predefined), since we supported the parents to maximize their infants’ self-initiated activity and make training part of the daily routine in the everyday environment. Small Step is a parent-led intervention, and whether or not this approach has any advantages over therapist-led training has still not been thoroughly investigated. We found it important to use parents as training providers for several reasons. The first consideration was to maintain training intensity, which can only be done by parents as part of everyday life in this age group of children. There was also a future-oriented perspective: Our aim was to strengthen the parents and make them feel that they were the ones in charge, knowing what was best for their child’s development, and were competent to offer support even if functional limitations were present. For upper extremity training, results do not differ depending on whether parents or therapists are the training providers when parents receive sufficient training and support [37].

From the parents’ perspective, it is traumatic to have an infant with a high risk of neurodevelopmental disorder, and it is well known that this affects parental well-being and mental health [38]. The mothers’ well-being seemed fairly stable over the intervention period, but there were many missing data, especially in the Standard Care group at the end of the study period and even more so at the follow-up when children were 2 years old. Data on social risk factors were unavailable in this study, but it can be noted that the educational level of the parents was very high, the mothers’ average age was over 32 years, and the living circumstances were acceptable according to the Swedish conditions.

An obvious limitation was that we could not measure the communication skills at baseline, as relevant standardized assessment tools for young infants are lacking [10]. Consequently, we could not find an assessment to use as the primary outcome measure that covered communication as well as gross and fine motor development. Yet, at 2 years of age, the BSDI III was used, but revealed no difference in the language domain between the two groups. We also included other communication assessments [17], but ultimately found one to be invalid: parents repeatedly reported problems understanding the Swedish Early Communicative Development Inventory questionnaire, so we stopped using it for data collection. Another assessment (i.e., the Responsive Augmentative and Alternative Communication Style Scale) was found to be unreliable and needs further development before it can be used for this age group of infants. We collected video recordings and will evaluate them using the Parent–Child Early Relationship Assessment in a future publication. There is also a plan for further publications including analysis following up the periodic intervention design of different foci as well as parental well-being. 

## 5. Clinical Implications and Conclusions 

The concept was found to be feasible and the included parents were very satisfied. We found that the most severely affected children benefited the most, indicating that the Small Step Program seems to be protective for such children. Small Step is also suitable for infants independent of their specific neurodevelopmental disorder due to its individualized approach, focusing on the children’s strengths and interests, and its support for families.

## Figures and Tables

**Table 1:** Baseline demographic characteristics of the participants (*n* = 39).

	Small Step (*n* = 19)	Standard Care (*n* = 20)	*P* Value
**Child Characteristics**			
Gestational age, weeks, mean (SD)	33 (6.5)	33 (6.95)	*p* > 0.05 ^a^
Inclusion CA age (T0), months, mean (SD) CA age at end of treatment (T5), mean (SD)	6.3 (1.62) 16.7 (2.23)	6.7 (1.96) 16.5 (1.96)	*p* > 0.05 ^a^
Gender, male/female	12/7	14/6	*p* > 0.059 ^b^
**CP risk factors**			
Extreme premature/preterm/term	6/5/8	8/3/9	*p* > 0.059 ^b^
AIMS (raw score) (T0)	18.0 (6.87)	19.52 (7,58)	*p* > 0.05 ^c^
HINE (T0) Neurological signs Behavior	49.0 (11.03) 13.8 (0.91)	48.27 (12.76) 14.3 (1.80)	*p* > 0.05 ^c^
Twins, *n* (families)	4 (3)	3 (2)	
MRI, available	13	16	
**Treatment**			
Therapist-led treatment hours, mean	28	16 range 0–58	
Weeks included in study (T0–T5), mean (SD)	43.5 (5.88)	41.7 (5.23)	*p* > 0.05 ^c^
**Mother’s characteristics** Age, years	32.7 (4.49)	34.7 (5.72)	*p* > 0.05 ^c^
First-born child	10	10	
HADS, T0, frequency, ^d^ depression/no depression (missing)	7/11	3/12 (4)	
HADS, T0, frequency, anxiety/no anxiety (missing)	8/10	3/12 (4)	
HADS, total mean, SD	13.5 (8,53)	10.4 (7.0)	
**Family socioeconomic factor** Education of 1 parent beyond ≥12 years	14	14	

Abbreviations: CP = cerebral palsy; CA = Corrected age for children born before week 37, extreme premature <29 weeks, preterm 29–36 weeks; AIMS = Alberta Infant Motor Scale, HINE = Hammersmith Infant Neurological Examination; HADS = Hospital Anxiety and Depression Scale, ^a^ Wilcoxon, ^b^ Chi-square, ^c^ two-sample *t*-test, ^d^ using the cut-off of 8.

**Table 2:** Means and 95% confidence intervals for the Peabody Developmental Motor Scales^2ed^ (PDMS, raw score), Gross Motor Function Measure-66 (GMFM-66), Pediatric Evaluation of the Disability Inventory (PEDI, functional scale, scaled score), and Bayley Scales of Infant Development III (BSID, index score).

Assessment	Small Step, *n* = 19			Standard Care, *n* = 19		
	T0	T5	2 Years	T0	T5	2 Years
PDMS, Stat	21.9 (18.8–25.0)	35 (32.7–37.3)	39.22 (31.1–47.4)	23.5 (20–26)	33.8 (31–36.6)	34.9 (32–37.9)
PDMS, Loc	19.7 (15.8–23.6)	58 (45.1–70.9)	70.83 (56.1–85.6)	20.4 (16.3–24.4)	58.8 (46.7–71.0)	71.7 (58.34–85.12)
PDMS, Gr	17.5 (13.3–21.7)	36 (32.6–39.4)	38.94 (35.6–42.25)	17.89 (13.9–21.9)	34.78 (30.2–39.4)	37.47 (32.6–42.3)
PDMS, Vm	20 (14.4–25.6)	59.6 (50.5–68.2)	72 (62.7–81.3)	20.68 (16.7–24.7)	53.89 (43.2–64.6)	71.15 (58.1–84.2)
GMFM-66	27.7 (24.8–30.5)	48.4 (43–54)	52.9 (45.4–60.3)	29.2 (26.5–31.8)	48.6 (43.6–53.7)	54.0 (47.5–60.5)
PEDI, SC		36.4 (32.7–40.2)	32.2 (27.3–37.1)		37.7 (34.9–40.6)	27.2 (19.8–34.6)
PEDI, Mob		43.2 (41.2–45.2)	47.9 (40.9–54.9)		37.7 (34.9–40.6)	44.5 (33.7–55.2)
PEDI, Soc		24.67 (19.24–30.1)	43.2 (41.2–45.1)		21.11 (13.2–29)	41.2 (35.9–46.8)
BSID, Cog			84 (73–96)			81 (70–92)
BSID, Lang			82 (75–89)			82 (73–91)
BSID, Mot			74 (61–87)			75 (65–83)

Stat = Stationary, Loc = locomotion, Gr = grasp, Vm = visuomotor integration, SC = self care, Mob = mobility, Soc = social function, Cog = cognitive development, Lang = receptive and expressive language development, Mot = fine and gross motor development.

**Table 3:** Final linear model analysis with PDMS-2 as the outcome at post intervention (T5) and at 2 years of age (T6). The models for secondary outcomes, GMFM-66, and the PEDI mobility scale are also reported.

	T5, After Intervention				T6, 2-Year Follow-Up			
	Mean PDMS-2		GMFM-66 *		Mean PDMS-2		PEDI Mobility	
	Reg. Coeff.	*P*-Value	Reg. Coeff.	*P*-Value	Reg. Coeff.	*P*-Value	Reg. Coeff.	*P*-Value
Small Step/Standard Care	18.271	**0.02**	18.977	**0.05**	21.497	**0.02**	15.666	**0.03**
PDMS-2, T0	1.525	**<0.001**	0.929	**0.01**	1.403	**0.016**	1.284	**<0.001**
HINE, T0	0.289	**0.04**	0.208	**0.04**	0.297	0.09	0.231	0.07
Time, T0–T5	0.416	0.07	0.251	0.13	0.159	0.576	0.0297	0.89
Inclusion age	–1.532	0.16	–0.830	0.33	–1.954	0.167	–2.420	**0.02**
GMFCS/2 groups	−9.522	0.26	−13.305	**<0.001**	−15.540	**0.01**	−17.482	**<0.001**
**Interaction**: Small Step/Standard Care and PDMS-2 T0	−0.839	**0.02**	−0.621	**0.06**	−1.095	**0.02**	−0.750	**0.02**

* When GMFM-66 was used at T5, PDMS-2 was replaced with GMFM-66 at T0 in the mode.

**Table 4:** Diagnostic information on participants, collected at 2 years of age.

	Small Step (*n* = 19)	Standard Care (*n* = 19)
**Diagnosis**		
Bilateral CP	5	6
Unilateral CP	0	1
Dyskinetic CP	3	2
Ataxic CP	1	0
Unspecific CP	1	1
Malformation syndrome	1	0
Autism spectrum disorder	1	0
Other neurodevelopmental disorder	6	8
Delayed development	1	0
Typical development	1	1
**Comorbidity**		
Epilepsy	6	2
Visual impairment	3	4
Hearing impairment (cochlea, *n* = 1)	1	2
Hydrocephalus, shunt	3	3
High comorbidity *	6	3
**Neuroradiological findings**		
Grey matter injury	1	2
White matter damage of immaturity	5	7
Maldevelopment	2	2
Miscellaneous	4	0
No visual deviation	1	5
Missing	6	3
**Mini-MACS**		
Level 1:2	8/2	12/3
Level 3:4:5	6/0/3	2/1/2
**GMFCS**		
Level 1:2	5/7	13/1
Level 3:4:5	4/1/2	2/1/2

* High comorbidity defined as three or more co-diagnoses.

**Table 5:** The following questions were included in the parental questionnaire concerning the feasibility and acceptability of the program.

Questions (Scored 1–9) *	Small Step (*n* = 17)	Standard Care (*n* = 8)
Based on your experience of the intervention, how useful do you find this kind of treatment?	8.6	6.4
To what extent do you think the intervention/follow-up has affected the pace of your infant’s development?	8.2	6.5
What is your overall experience of the attitude and approach of the staff towards you and your family?	8.6	9
How important was answering the questions on parental wellbeing?	6.7	6
How useful were the hospital assessments of your child?	8.3	7.5
Do you feel you received sufficient feedback from us on the assessments of your child’s development?	7.6	7.5
How likely is it that you would recommend this treatment to a friend with an infant with developmental delay?	8.8	
Rate your motivation to participate in this training programme.	8.3	
During treatment, training was conducted for one developmental domain at a time: gross motor skills, communication, and fine motor skills. To what extent do you think that training each domain separately was an advantage (high value) or a disadvantage (low value)?	8.1	
You met several staff with different backgrounds, knowledge, and expertise. To what degree do you rate this as an advantage (high value) or a disadvantage (low value)?	8.2	

* 1 = using words such as: not at all, not good, not everyone, and disadvantage; 9 = using words such as: to a large extent and advantage.

## Supplementary Materials

Supplementary Tables S1 and S2. Characteristics of participating children diagnosed with CP or other neurological disorders at two years of age. Available online at https://www.mdpi.com/2077-0383/8/7/1016/s1.
