# Supplementary material for: Efficacy of the Small Step Program in a Randomized Controlled Trial for Infants under 12 Months Old at Risk of Cerebral Palsy (CP) and Other Neurological Disorders
Source: J Clin Med. 2019 Jul 11;8(7):1016. doi: 10.3390/jcm8071016 (PMC6679038; doi:10.3390/jcm8071016)
Supplement: Supplementary file 1 [file jcm-08-01016-s001.pdf]

Journal of Clinical Medicine

Supplementary Table S1. Characteristics of participating children diagnosed with CP at two years of age.

Group	ID	GA	Diagnosis	MRI	PDMS, range of age equivalent months	GMFCS - E&R
**SS** [a]	10	40	CP ataxic	Miscellaneous	11–24	3
**SS**	1	40	CP dyskinetic	GMI [c]	3–4	5
**SS**	14	40	CP dyskinetic	Miscellaneous	3–5	5
**SS**	30	26	CP dyskinetic	VMDI [d]	5–12	4
**SC** [b]	19	40	CP dyskinetic	No MR	1–4	5
**SC**	29	25	CP dyskinetic	Miscellaneous	11–15	1
**SC**	7	31	CP bilateral	VMDI	15–34	3
**SC**	9	40	CP bilateral	VMDI	9–19	3
**SC**	16	25	CP bilateral	VMDI	14–23	1
**SC**	17	25	CP bilateral	VMDI	17–24	1
**SS**	2	27	CP bilateral	VMDI	8–15	4
**SS**	22	26	CP bilateral	VMDI	7–14	3
**SS**	25	40	CP bilateral	VMDI	11–18	2
**SS**	28	38	CP bilateral	VMDI	11–24	2
**SC**	21	24	CP bilateral	VMDI	11–21	1
**SC**	6	40	CP bilateral	GMI	3–8	5
**SS**	38	29	CP bilateral	–	14–24	2
**SC**	20	40	CP unilateral	GMI	16–24	2
**SC**	34	26	CP unspecified	VMDI (unclear)	4–9	3
**SS**	23	23	CP unspecified	No MR	5–13	3

[a] SS=Small step, [b] SC= standard Care, [c]GMI = grey matter injury, [d] WMDI=white matter damage of immaturity.

Supplementary Table S2. Characteristics of participating children diagnosed with other neurological disorders at two years of age.

Group	ID	GA	Diagnosis	MRI	PDMS, range of age equivalent months	GMFCS
SC	31	24	Autism	No MRI	13–17	1
SS	32	35	Autism	No MRI	18–37	2
SS	18	31	Maldevelopment	Maldevelopment	15–22	1
SC	3	40	Other neurological disorder	Maldevelopment	12–18	1
SC	13	40	Other neurological disorder	No visual deviation	18–24	1
SC	15	33	Other neurological disorder	No MRI	13–18	1
SC	26	30	Other neurological disorder	No visual deviation	16–24	1
SC	27	40	Other neurological disorder	No visual deviation	11–18	1
SC	35	37	Other neurological disorder	No MRI	17–24	1
SC	37	23	Other neurological disorder	WMDI/no visual deviation	11–21	1
SS	4	37	Other neurological disorder	Maldevelopment	12–15	2
SS	8	40	Other neurological disorder	Maldevelopment	9–13	2
S	11	40	Other neurological disorder	No MRI	18–24	1
S	24	23	Other neurological disorder	No visual deviation	18–24	1
S	36	31	Other neurological disorder	No MRI	13–22	2
S	12	26	Slightly delayed	No MRI	13–22	1
S	33	34	No diagnosis	Miscellaneous	14	1
C	39	27	No diagnosis	No visual deviation	20–24	1
